# Coupled Plasma-Catalytic System with Rang 19pr Catalyst for Conversion of Tar

**DOI:** 10.1038/s41598-019-49959-4

**Published:** 2019-09-19

**Authors:** Michał Młotek, Joanna Woroszył, Bogdan Ulejczyk, Krzysztof Krawczyk

**Affiliations:** Warsaw University of Technology, 3 Noakowskiego Street, 00-664 Warsaw, Poland

**Keywords:** Heterogeneous catalysis, Pollution remediation

## Abstract

A coupled plasma-catalytic system (CPCS) for the conversion of toluene was investigated and compared to the homogeneous system of gliding discharge plasma. Toluene was used as a model compound, which is present in tars. The study was carried out at atmospheric pressure, in a gas composition similar to the one obtained during pyrolysis of biomass. The effect of the initial toluene concentration, energy supplied to gliding discharge (GD) and the presence of a catalyst on the conversion of toluene was studied. Both the composition of outlet gas and its calorific value were monitored. Based on the obtained results it can be concluded that the conversion of toluene increases with the increase of gliding discharge power. The highest toluene conversion (89%) was received in the coupled plasma-catalytic system (catalyst: RANG-19PR) under the following conditions: CO (0.13 mol. fr.), CO_2_ (0.12 mol. fr.), H_2_ (0.25 mol. fr.), N_2_ (0.50 mol. fr.) and 4400 ppm of toluene with a gas flow rate of 1000 Nl/h. The composition of the outlet gas in the homogeneous system and in the CPCS changed in the range of a few percents. Toluene levels were reduced tenfold. Benzene, C3 and C4 hydrocarbons, as well as acetylene, ethylene and ethane, were detected in the outlet stream in trace amounts. Carbon deposits were present in the reactor. The products of methanation of carbon oxides were detected in the both studied systems. A mechanism of toluene decomposition in the CPCS was proposed. The application of the catalyst brought about an increase in the calorific value of the outlet gas. It was above the minimal level demanded by engines and turbines.

## Introduction

Biomass is currently considered the most promising source of renewable energy. Technologies for efficiently harvesting the energy contained in biomass are rapidly developed^1,2^. The two most common processes applied in transforming the chemical energy contained in biomass into useful energy are pyrolysis and gasification. In these processes, gaseous products are formed. They mainly contain hydrogen, hydrocarbons and carbon oxides^3^. Such gas can be used in the chemical synthesis or to drive engines and turbines. However, there are restrictions to the applicability of the pyrolysis gas obtained from biomass due to the presence of tars, mainly mono and polycyclic aromatic hydrocarbons. Their amount strongly depends on the type of biomass. Their concentration can vary between 5 and 18 g/Nm^3^ ^4–6^. Such mixtures do not meet requirements for engines or turbines and require further purification^2,7^. The number of tars in gas used as a fuel for engines must not exceed 50–100 mg/Nm^3^ and 5 mg/Nm^3^ for turbines^7,8^. Therefore, before the obtained gas can be utilized, it must be purified. Tars present in the gas after biomass gasification may deposit on reactor walls, corrode it and obstruct the flow, which reduce the process efficiency^2^ and causes a damage of engines and turbines.

There are many technologies used for the purification of pyrolysis gas from volatile organic compounds and tars. The following methods can be distinguished: catalytic oxidation, filtration and biofiltration, adsorption on activated carbon, electrostatic precipitation and plasma techniques^8–12^. Decomposition of tars has been conducted with the use of natural and synthetic materials. Each of them has advantages and disadvantages. For example, minerals are usually cheap, but they are easily eroded. The iron ore is reduced under the influence of gas components, whereas clays are decomposed at high temperatures. Synthetic catalysts lead to a higher conversion of tars^7^, but they are more expensive and more susceptible to sulfur deactivation and soot contamination. Nowadays, the most common catalytic systems used and investigated for tar decomposition are Ni, Rh, Pt, Fe based catalysts^7,12^. Some literature results on the destruction of model compounds with a catalyst or the use of plasma are presented in Table 1. The conversion of tars depends on the compound used as the model in the studies, as well as the catalyst and temperature of the process^1,2,7–15^. A homogeneous non-thermal plasma and a CPCS have been widely used for many environmental technologies^16–18^. The conversion of toluene also has been investigated in a homogeneous plasma system^19,20^ and a CPCS^21^. However, all of these studies show that high temperature is required and none of them led to the development of an effective method of purification of the gas after pyrolysis, which is beneficial on an industrial scale. The new articles concerning a plasma-catalytic decomposition of tar in synthetic gas after pyrolysis or steam reforming of tar were published^22–26^. In these studies, gliding discharge (GD) or dielectric barrier discharge (DBD) reactors were used. It was found that the conversion of tar compound and product selectivity depends on the process condition and input energy. During the chemical changes and radical reactions in plasma conditions, many different compounds may be formed. Toluene can be decomposed to carbon dioxide or to oxygenates like benzoic acid or phenol^23^. The increase of yield and selectivity of the decomposition of tars can be reached by the use of the active catalyst.

**Table 1:** Collection of literature results of catalytic and plasma decomposition of model tar compounds. GD - gliding discharge, E.C. - energy consumption [g/kWh].

Catalyst	Conditions	Model compound	Conc.[%]	Conv.[%]	E.C.[g/kWh]	Ref.
ICI 46-1 (Ni catalyst)	700–900 °C	Benzene Toluene Anthracene Naphthalene Pyrene	n/a	60–80 40–80 40–90 20–80 30–90	n/a	^9^
Co-Fe/α-Al_2_O_3_	Steam reforming 600 °C	Benzene Toluene	1.4 1.2	40 46.2	n/a	^10^
Co/α-Al_2_O_3_		Benzene Toluene	1.4 1.2	25.2 56.9	n/a	
Ni/Olivine	650 °C	Toluene	n/a	100	n/a	^13^
Olivine	850 °C	Toluene	n/a	37	n/a	
Ni/MgO/Olivine	800 °C	Toluene	n/a	100	n/a	^14^
None (steam addition)	GD plasma	Benzene Naphthalene	1.4–7 0.6–1.3	95 79	120 68	^19^
None (air addition)	GD plasma	Toluene Naphthalene	0.6–2.3 0.1–0.5	90 70	215–796 62.5–206	^20^
industrial catalyst for methane steam reforming G-0110	880 °C	Toluene	n/a	84	n/a	^21^
	GD plasma 880 °C	Toluene	n/a	89	n/a	
Ni/γ-Al_2_O_3_	DBD plasma 400 °C	Toluene	0.06	~100	25,7	^24^

Therefore, the investigation of the CPCS of gliding discharge with a spherical, commercial nickel catalyst (RANG 19PR), allowing to operate at lower temperatures with a high toluene conversion, is beneficial and can be considered for an industrial scale. The effect of the application of CPCS, in which a series of reactions takes place, on the calorific value of the obtained gas was also studied.

## Experimental

**Figure 1:**
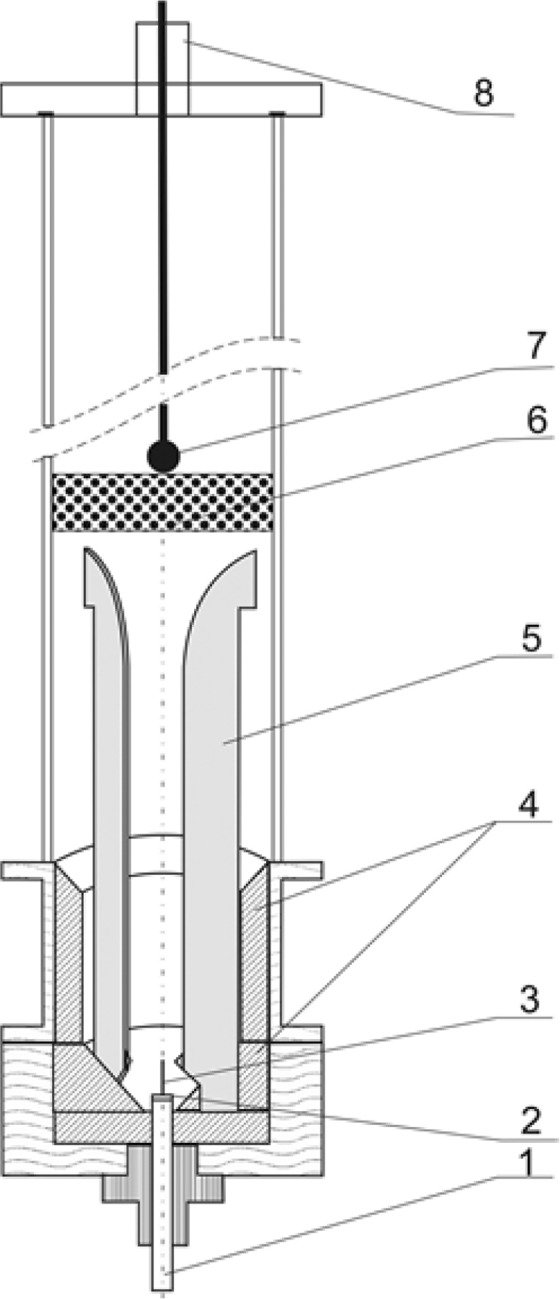
Three-phase gliding discharge reactor. 1 – gas inlet, 2 – gas nozzle, 3 – starting electrode, 4 – ceramic lining, 5 – electrode, 6 – bed of catalyst, 7 – thermocouple, 8 – gas outlet^27^.

Toluene was used as a model compound, which could represent tars from biomass. The conversion of toluene in a three-phase gliding discharge reactor (Fig. 1) under atmospheric pressure was investigated. The reactor, described previously in^27^, was made of quartz–glass tube of 60 mm inner diameter and was equipped with three converging duralumin electrodes. The gas inlet was located at the bottom, between the electrodes. The nozzle area was 1.5 mm^2^ and it consisted of three nozzle outlets of 0.8 mm in diameter. The temperature of the outlet gas was measured by a thermocouple at the vessel axis, 10 mm over the upper ends of the electrodes. The gliding discharge did not reach the end of the thermocouple. When the CPCS system was used the end of the thermocouple was placed in the middle of the catalyst bed.

The power supply consisted of ferroresonant transformers and a transistor inverter, which allowed us to obtain a poly–phase multi-electrode reactor system. The electric power source was made by Ertec-Poland^28^. Electric power of a gliding discharge reactor was in the range of 1–2 kW. It was measured by an energy meter. The gases flow rate was controlled by Bronkhorst gas flow meter. Toluene was introduced into the gas stream by thermostated bubbling vessel.

During the selection of the catalyst for the CPCS, the results of our previous studies and data from the literature concerning the conversion of tars were taken into account^29^. The industrial spherical catalyst for carbon oxides methanation, Ni + NiO/Al_2_O_3_ (RANG 19PR), was used for the experiments. The industrial catalyst for methanation of carbon oxides was used due to its repetitive parameters, low price and resistance to the formation of carbon deposits on its surface, which is especially important in the tar decomposition and deactivation of the catalysts. This catalyst is also stable during methanation of carbon oxides. To obtain catalyst particles suitable for research, the industrial catalyst spheres with a diameter of 5 mm were crushed and the fraction 1.6–3.15 mm was used. The catalyst was packed into the reactor without additional pretreatment. The catalyst particles were packed into the reactor in a single batch of 40 ml approx. 1.5 cm high. The distance between catalysts and electrodes was about 7 mm.

The gas temperature reached 160–400 °C and exhibited a dependence on the discharge power and the presence of a catalyst. The process of toluene decomposition was tested at a gas flow rate of 1000 Nl/h. Tests in the homogeneous system were conducted in a wide range of initial concentrations of toluene of 2000–4400 ppm, 8.8–19.3 g_toluene_/Nm^3^ respectively. For further studies with the CPCS, the initial concentration of toluene 3200 ppm (14 g_toluene_/Nm^3^) was chosen.

The composition of used gas (in the molar fraction) was CO (0.13), CO_2_ (0.12), H_2_ (0.25), N_2_ (0.5), the humidity did not exceed 1% when measured at 25 °C. For the gas analysis, two kinds of gas chromatographs were used. First Thermo-Scientific Trace 1300 with an HP5 column and a Flame Ionized Detector (FID) to determine the toluene concentration and mass spectrometer (MS) for other higher than C5 hydrocarbons analysis. Second Agilent 6890 N with a ShinCarbon column and the following detectors: TCD and FID for the following gases: CO_2_, CO, H_2_, N_2_, O_2_, CH_4,_ and C2 hydrocarbons analysis. The water vapor content was measured by an APAR moisture meter^27^.

The calculations were made using the following equations:

1. Overall toluene conversion:
  $${\rm{x}}[{{\rm{C}}}_{7}{{\rm{H}}}_{8}]=\frac{{{\rm{W}}}_{0}[{{\rm{C}}}_{7}{{\rm{H}}}_{8}]-{\rm{W}}[{{\rm{C}}}_{7}{{\rm{H}}}_{8}]}{{{\rm{W}}}_{0}[{{\rm{C}}}_{7}{{\rm{H}}}_{8}]}$$
2. Energy consumption of toluene decomposition:
  $${UEC}[{{C}}_{7}{{H}}_{8}]=\frac{{{W}}_{0}[{{C}}_{7}{{H}}_{8}]\times x[{{C}}_{7}{{H}}_{8}]\times {M}[{{C}}_{7}{{H}}_{8}]}{{P}}$$
3. The calorific value of the fresh and outlet gas:

$${W}=\frac{{{Q}}_{{p}{{H}}_{2}}\cdot {{a}}_{{{H}}_{2}}+{{Q}}_{{pCO}}\cdot {{a}}_{{CO}}+{{Q}}_{{pC}{{H}}_{4}}\cdot {{a}}_{{C}{{H}}_{4}}+{{Q}}_{{p}{{C}}_{2}{{H}}_{2}}\cdot {{a}}_{{{C}}_{2}{{H}}_{2}}+{{Q}}_{{p}{{C}}_{2}{{H}}_{4}}\cdot {{a}}_{{{C}}_{2}{{H}}_{4}}+{{Q}}_{{p}{{C}}_{2}{{H}}_{6}}\cdot {{a}}_{{{C}}_{2}{{H}}_{6}}}{1000}$$

where:

x[C_7_H_8_] – overall conversion of toluene;

W_0_[C_7_H_8_], W[C_7_H_8_] – toluene flow rate at the inlet and outlet respectively [mol·h^−1^],

M[C_7_H_8_] – toluene molar mass – 92.14 [g·mol ^−1^].

P – discharge power [kW]

W – calorific value [MJ/m^3^]

Q_p_ – the heat of combustion [kJ/m^3^]

a – molar fraction

The morphology of the catalyst’s surface was tested using Quanta 3D FEG (Thermo Fisher Scientific) scanning electron microscope equipped with detectors of secondary (SE) and backscattered (Zcont) electrons and an Apollo X energy dispersive x-ray spectrometer (EDAX). The X-ray diffraction (XRD) analysis was carried out with a Seifert 3003 diffractometer using CuKα radiation^27^.

## Results and Discussion

### Studies of the catalyst surface

**Figure 2:**
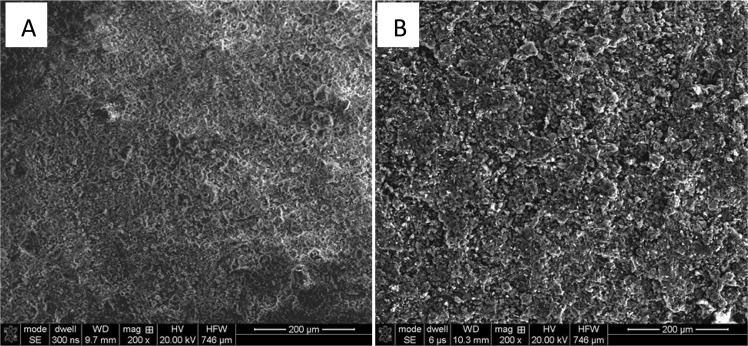
The morphology of the RANG 19PR catalyst surface. Before measurements (**A**), after measurements (**B**).

**Figure 3:**
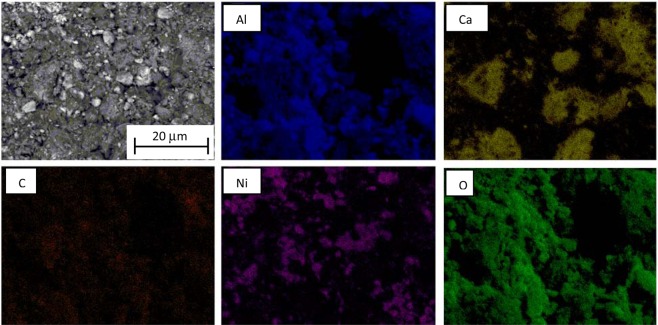
The SEM image with EDX elemental analysis maps of the external surface of the *RANG-19PR* before measurements.

**Figure 4:**
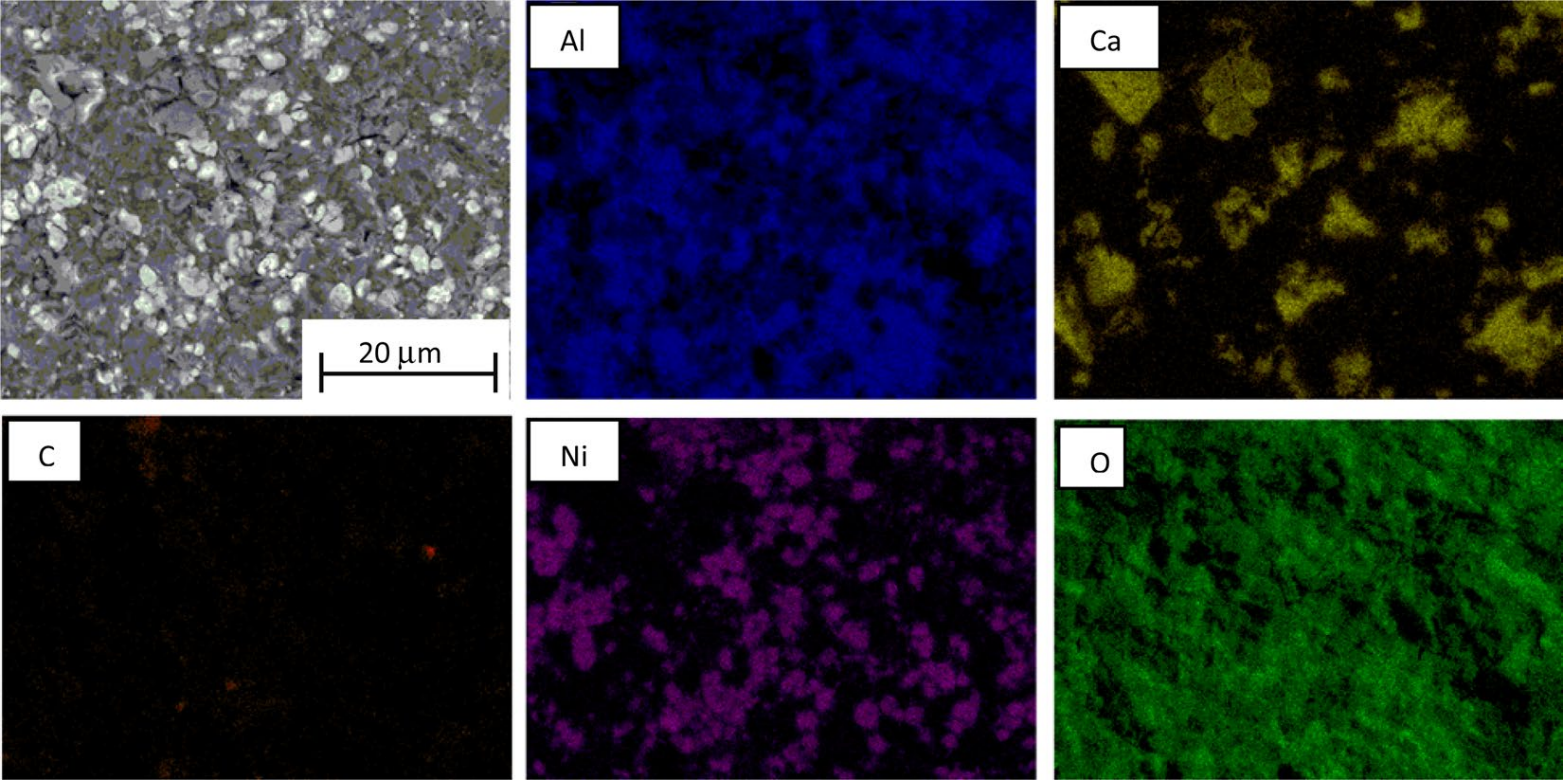
The SEM image with EDX analysis of the external surface of the fresh *RANG-19PR* catalyst after measurements.

**Figure 5:**
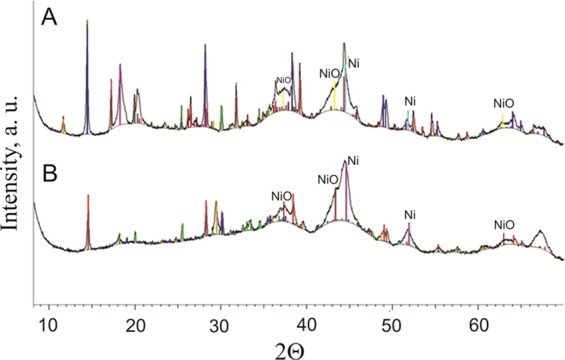
XRD spectra of the fresh catalyst: (**A**) before, and (**B**) after measurements.

RANG-19PR is the commercial catalyst for carbon monoxide methanation obtained in the co-precipitation process. The fresh form of catalyst consists of Ni, NiO, Al_2_O_3_ and high-alumina cement. The concentration of both forms of Ni was 19 wt%^30,31^. The reduced form of RANG 19PR contains only Ni without NiO. The surface of the catalyst calculated from the BET absorption isotherm was 152 m^2^/g before and 117 m^2^/g after the reaction. The composition of the catalyst also changed during the activity measurements. The texture of the surface changed (Fig. 2). The surface was more roughened and a substantial amount of carbon was formed as seen in the EDX maps (Figs 3 and 4). Nickel oxide and metallic nickel were present both before and after the measurement. The existence of Ni pure or in oxide form on the surface was confirmed by XRD analysis (Fig. 5). The EDX microanalysis of catalyst surface showed nickel as the basic element, which is the main component of the catalyst. The distribution of it on carrier surface is uniform, however, in some areas, nickel absence can be seen. EDX analysis showed also a presence of aluminum and calcium. These substances are the components of the very complicated and patented structure of catalyst carrier (Fig. 5). Some carbon can be seen on the catalyst surface (Figs 3, 4). In the fresh catalyst, carbon was from carbonates from the carrier. Carbon observed using EDX analysis of catalyst after the process was mostly the carbon black deposit. This can be the reason of low increase of toluene conversion in CPCS.

### Effect of plasma and CPCS on gas composition

**Figure 6:**
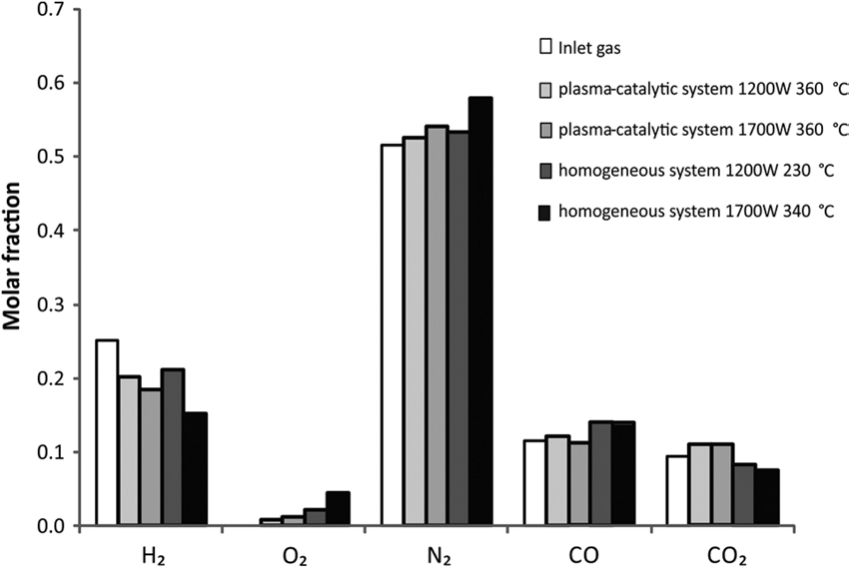
The molar fraction of H_2_, O_2,_ N_2_, CO, CO_2_ obtained in the CPCS (initial toluene concentration 3200 ppm).

The measurements of toluene decomposition were performed in a homogeneous system (plasma only) and a CPCS. In the CPCS a fixed bed of the nickel catalyst, RANG 19PR, was used. The following gases were detected: CO_2_, CO, H_2_, CH_4_, C2-C3 hydrocarbons, H_2_O and trace amounts of C4 hydrocarbons (Fig. 6).

**Figure 7:**
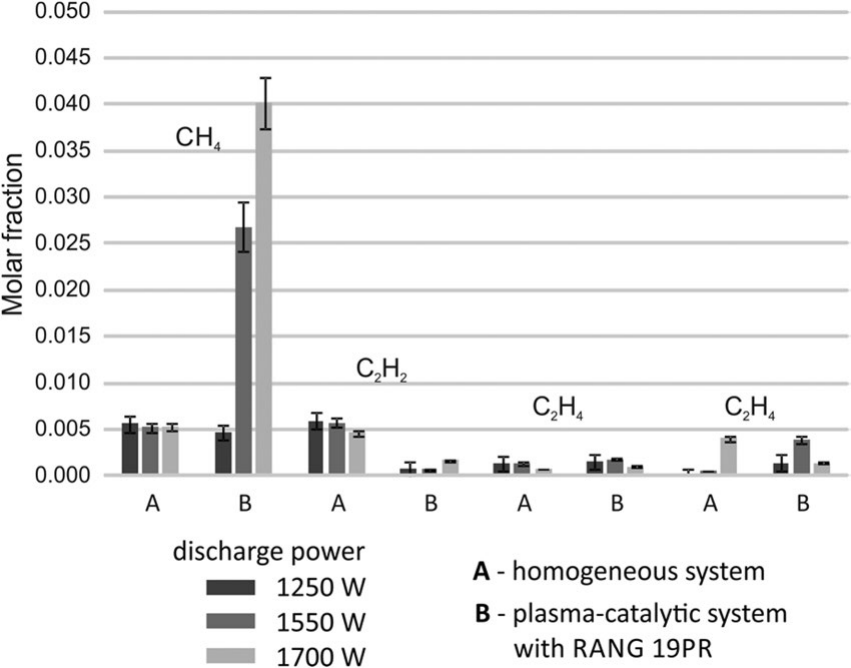
Molar fractions of methane and C2 hydrocarbons in the outlet gas after the plasma and plasma-catalytic process of toluene decomposition in a simulated post-pyrolysis gas.

In the CPCS, the molar fraction of hydrogen decreased with the discharge power regardless of the initial toluene concentration. The molar fraction of nitrogen was the same in the inlet and outlet streams, which indicates a small change in the volume of gases as a result of the process. Trace amounts of oxygen were formed in each of the studied systems. In the homogeneous system, the concentration of carbon dioxide was lower than in the case of CPCS. Moreover, the concentration of carbon oxide and oxygen was higher (Fig. 7). This could be the reason for carbon dioxide dissociation. In CPCS the concentration of carbon oxides did not change significantly. Oxygen concentration was much lower than in the homogeneous system. This could be the effect of methanation of carbon oxide and water formation from oxygen and hydrogen on the catalyst’s surface.

In the CPCS significant quantities of methane were observed in the outlet gas stream. Methane could be formed in two processes: decomposition of toluene and methanation of carbon oxides. In the homogeneous system, the concentration of methane did not depend on the discharge power and temperature. This suggests that both reactions, in which methane was generated, can run in parallel. However, in the CPCS the methane concentration rapidly increased with an increase of discharge power. The rise of discharge power caused a rise of temperature, which resulted in an increase of exothermic reaction rate of methanation on a catalyst surface. RANG-19PR is the industrial methanation catalyst. It is active above 200 °C so the high concentration of methane at 1750 W resulted mainly from the methanation of carbon oxides. In the outlet gases, the trace amounts of C2 hydrocarbons were detected. The presence of these gases was the effect of toluene decomposition (ethyne mainly)^32^ and methane coupling (Fig. 7)^33^.

The humidity of the outlet gas was in range 60 and 90%, measured at 25 °C, and strongly depended on temperature and not on discharge power. At the temperature 360–400 °C and discharge power of 1200 and 1700 W water content was constant. It was 90%. However, at constant discharge power (1250 W) and temperatures, 170 and 400 °C humidity of the gas was 60 and 90% respectively. It can be concluded that water was the product of catalytic carbon oxide methanation and not of the radical reaction of hydrogen and oxygen.

### Effect of plasma and CPCS on toluene conversion

**Figure 8:**
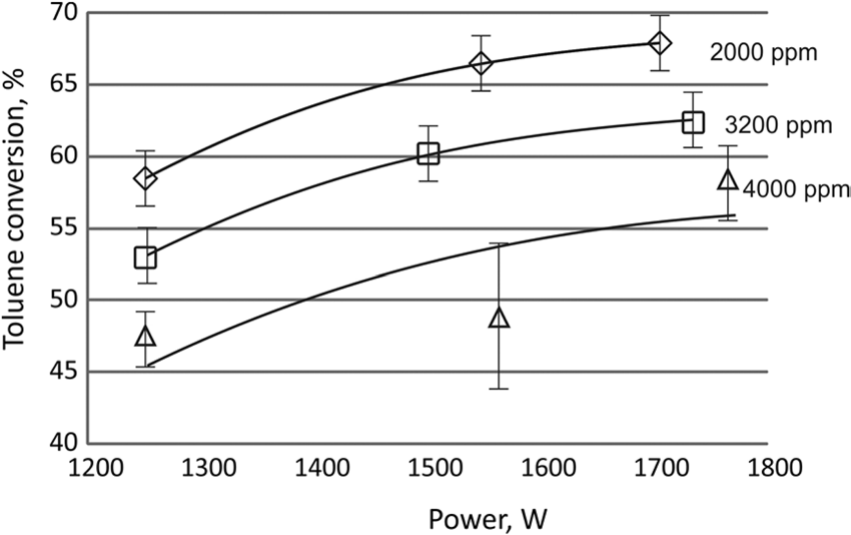
Effect of power of gliding discharge on toluene conversion in the homogeneous system. The initial concentration of toluene 2000, 3200 and 4400 ppm.

The effects of the power and the initial concentration of toluene on its conversion were also studied in the gliding discharge reactor. The highest conversion, i.e. 68%, was achieved at the lowest initial concentration of toluene (2000 ppm). On the basis of the obtained results, it was found that the toluene conversion decreases with the increase of the initial concentration of toluene in the inlet gas and increases with increasing discharge power (Fig. 8, Table 2). It was observed that in the CPCS the amount of carbon oxide and hydrogen was smaller than in the homogeneous system. Moreover, methane was detected in the outlet gas mixture. The highest conversion of toluene was obtained with the CPCS with the initial toluene concentration of 4400 ppm.

**Table 2:** Toluene conversion in the homogeneous system and the CPCS.

Initial concentration of toluene	Discharge Power [W]	Homogeneous system		CPCS	
		Toluene conversion [%]	Temperature [°C]	Toluene conversion [%]	Temperature [°C]
2000	1250	58.5	260	29.7	190
	1550	66.5	330	63.3	260
	1700	67.9	370	75.9	320
	1250	—	—	—	—
3200	1300	53.0	230	49.2	170
	1500	60.2	290	61.1	230
	1700	62.4	340	81.7	360
	1250	—	—	72.3	400
	0	—	—	37.1	360
4400	1250	43.1	250	20.6	160
	1550	48.0	320	73.2	280
	1700	58.8	360	89.3	360
	1250	—	—	77.9	400
	0	—	—	46.1	360

**Figure 9:**
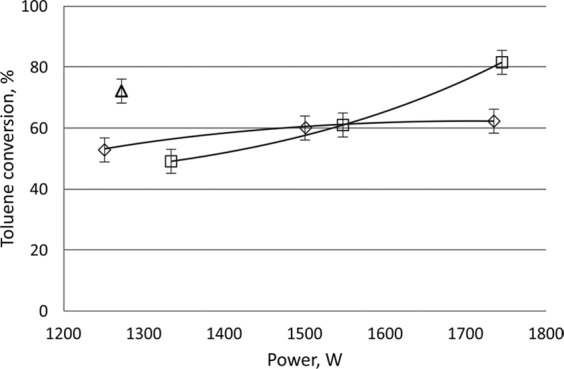
Effect of power on toluene conversion. 3200 ppm of toluene. Gas flow rate 1000 Nl/h. ◇ - homogeneous system, □ - plasma catalytic system, △ - plasma catalytic system at 400 °C.

When using 1250 W discharge power at high temperature (reached by the conducting the process at 1700 W discharge power), the conversion in the CPCS was similar to those obtained with 1550 W discharge power. After turning off the discharge, the temperature of the gas rapidly decreased and the conversion of toluene increased (Fig. 9, Table 2). As the result of heating of the catalyst, the conversion of toluene using a discharge power 1300 W was similar to that observed with the 1700 W discharge power (Fig. 9).

**Figure 10:**
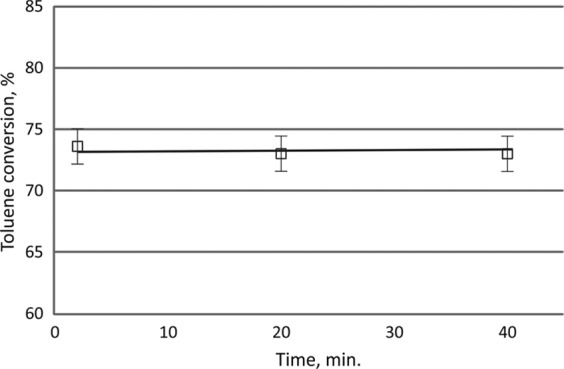
Toluene conversion during the process for 40 minutes. The initial concentration of toluene 3200 ppm, discharge power 1550 W.

In the homogeneous system, methane and trace amounts of acetylene, ethylene, and ethane were formed. In CPCS C2 hydrocarbons were also detected, however, their concentration was also very low. RANG-19PR catalyzed methanation of carbon oxides. Therefore, a substantial amount of methane was formed. Its concentration increased with increasing discharge power. The catalyst was stable in the CPCS for 40 minutes (Fig. 10). This study was conducted at initial toluene concentration 3200 ppm and discharge power 1500 W. Under these conditions energy efficiency was 6.7 g_toluene_ /kWh. This value is in similar range to those presented in literature, i.e. 3.6 g/kWh^34^, 2.5 g/kWh^29^, 3–15 g/kWh^35^.

**Figure 11:**
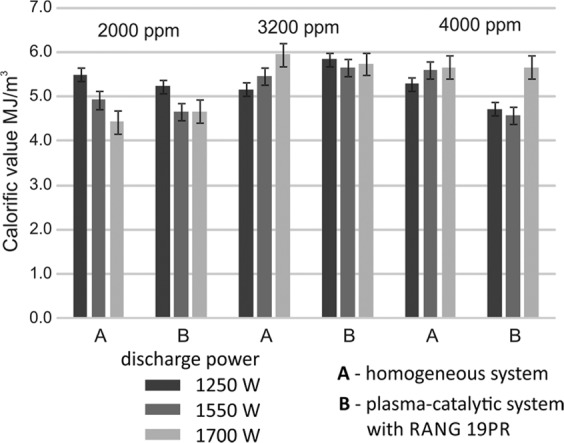
The calorific value of the outlet gas. Average inlet gas calorific value was 5 MJ/m^3^. Demanded level of calorific rate is 4 MJ/m^3^.

In the homogeneous system at a discharge power of 1250 W, the calorific value of the outlet gas decreased with the increase of the initial toluene concentration. However, at discharge power 1550 and 1700 W, the calorific value of the outlet gas increased with the increase of the initial toluene concentration (Fig. 11). It was the effect of the increase of carbon monoxide concentration caused by the carbon dioxide dissociation and toluene decomposition. In the CPCS with RANG-19PR catalyst at a discharge power of 1550 and 1700 W, the calorific value of the outlet gas increased at the initial toluene concentration 3200 and 4000 ppm compared to the initial toluene concentration 2000 ppm. The highest calorific value of the outlet gas was received in the homogeneous system and the CPCS at a discharge power of 1700 W at the initial toluene concentration 3200 and 4000 ppm. It was 5.95 and 5.66 in the homogeneous system and 5.74 and 5.66 in the CPCS, respectively (Fig. 11). For both investigated systems, it was possible to obtain the calorific value of the gas higher than the acceptable minimum level of 4 MJ/m^3^.

### Reaction mechanism chain

One of the first steps of the reaction in a mixture of carbon oxides, hydrogen, nitrogen and toluene consists in radical formation and attachment of an electron, resulting in the formation of C_6_H_5_, CH_3_ and of hydrogen radicals (reactions 1–3)^36–40^:

1 $${{\rm{C}}}_{6}{{\rm{H}}}_{5}{{\rm{CH}}}_{3}+{{\rm{e}}}^{\ast }\to {{\rm{C}}}_{6}{{\rm{H}}}_{5}+{{\rm{CH}}}_{3}+{\rm{e}}$$

2 $${{\rm{C}}}_{6}{{\rm{H}}}_{5}{{\rm{CH}}}_{3}+{{\rm{e}}}^{\ast }\to {{\rm{C}}}_{6}{{\rm{H}}}_{4}{{\rm{CH}}}_{3}+{\rm{H}}+{\rm{e}}$$

3 $${{\rm{C}}}_{6}{{\rm{H}}}_{5}{{\rm{CH}}}_{3}+{{\rm{e}}}^{\ast }\to {{\rm{C}}}_{6}{{\rm{H}}}_{5}{{\rm{CH}}}_{2}+{\rm{H}}+{\rm{e}}$$

Moreover, other gases present in a gas mixture may be also excited or dissociated by electron impact (reactions 4–6)^26^:

4 $${{\rm{N}}}_{2}+{{\rm{e}}}^{\ast }\to {{{\rm{N}}}_{2}}^{\ast }+{\rm{e}}$$

5 $${{\rm{CO}}}_{2}+{{\rm{e}}}^{\ast }\to {\rm{CO}}+{{\rm{O}}}^{\mathrm{..}}+{\rm{e}}$$

6 $${{\rm{H}}}_{2}+{{\rm{e}}}^{\ast }\to 2{\rm{H}}+{\rm{e}}$$

The excited molecules play the important role in a chemical reaction chain of toluene decomposition. These species react with toluene generating more toluene radicals (reactions 7–10) or recombine mainly reproducing the substances from which they obtained.

7 $${{\rm{C}}}_{6}{{\rm{H}}}_{5}{{\rm{CH}}}_{3}+{{{\rm{N}}}_{2}}^{\ast }\to {{\rm{C}}}_{6}{{\rm{H}}}_{5}+{{\rm{CH}}}_{3}+{{\rm{N}}}_{2}$$

8 $${{\rm{C}}}_{6}{{\rm{H}}}_{5}{{\rm{CH}}}_{3}+{\rm{H}}\to {{\rm{C}}}_{6}{{\rm{H}}}_{5}{{\rm{CH}}}_{2}+{{\rm{H}}}_{2}$$

9 $${{\rm{C}}}_{6}{{\rm{H}}}_{5}{{\rm{CH}}}_{3}+{\rm{H}}\to {{\rm{C}}}_{6}{{\rm{H}}}_{5}+{{\rm{CH}}}_{4}$$

10 $${{\rm{C}}}_{6}{{\rm{H}}}_{5}{{\rm{CH}}}_{3}+{{\rm{O}}}^{\mathrm{..}}\to {{\rm{C}}}_{6}{{\rm{H}}}_{4}{{\rm{CH}}}_{2}+{\rm{OH}}$$

The C_6_H_4_CH_3_ or C_6_H_5_CH_2_ radicals react with H radical restoring the toluene or with O^∙∙^ or OH radicals forming oxygenates like benzyl alcohol or aldehyde (reactions 11–12).

11 $${{\rm{C}}}_{6}{{\rm{H}}}_{5}{{\rm{CH}}}_{2}+{{\rm{O}}}^{\mathrm{..}}\to {{\rm{C}}}_{6}{{\rm{H}}}_{5}{{\rm{CH}}}_{2}{\rm{O}}$$

12 $${{\rm{C}}}_{6}{{\rm{H}}}_{5}{{\rm{CH}}}_{2}+{\rm{OH}}\to {{\rm{C}}}_{6}{{\rm{H}}}_{5}{{\rm{CH}}}_{2}{\rm{OH}}$$

However, taking into account the gliding discharge plasma condition and initial concentration of toluene the reactions 11 and 12 should have a low reaction rate. Moreover, under plasma conditions benzyl alcohol or aldehyde undergo subsequent reactions of hydrogenation or decomposition^23^. The C_6_H_5_ radicals may react with hydrogen^41^ to form soot precursors (reactions 13–14)^36^.

13 $${{\rm{C}}}_{6}{{\rm{H}}}_{5}+{\rm{H}}\to {{\rm{C}}}_{6}{{\rm{H}}}_{6}$$

14 $${{\rm{C}}}_{6}{{\rm{H}}}_{5}+{\rm{H}}\to 3{{\rm{C}}}_{2}{{\rm{H}}}_{2}+{\rm{e}}$$

The products of reactions 13 and 14 are benzene and ethyne. This is the intermediate step of toluene oxidation to CO_2_ and H_2_O. The intermediate products can also be decomposed to soot (reactions 15–16)^42^ and after that, oxidized to CO_2_.

15 $${{\rm{C}}}_{6}{{\rm{H}}}_{6}\to 6{\rm{C}}+3{{\rm{H}}}_{2}$$

16 $${{\rm{C}}}_{2}{{\rm{H}}}_{{\rm{n}}}\to 2{\rm{C}}+{\rm{n}}/2\,{{\rm{H}}}_{2}$$

In the CPCS methanation of carbon oxides (17), (18) or carbon black hydrogenation (19)^41–43^ can occur

17 $${\rm{CO}}+3{{\rm{H}}}_{2}\to {{\rm{CH}}}_{4}+{{\rm{H}}}_{2}{\rm{O}}$$

18 $${{\rm{CO}}}_{2}+4{{\rm{H}}}_{2}\to {{\rm{CH}}}_{4}+2{{\rm{H}}}_{2}{\rm{O}}$$

19 $$2{{\rm{H}}}_{2}+{\rm{C}}\to {{\rm{CH}}}_{4}$$

However methane can also be formed from methylene radicals (formed in reaction 1) and hydrogen radicals (20).

20 $${{\rm{CH}}}_{3}+{\rm{H}}\to {{\rm{CH}}}_{4}$$

Besides the methanation reactions (17) and (18), the carbon monoxide can also undergo the water–gas shift reaction (21) or can be formed in the Boudouard’s reaction (22).

21 $${\rm{CO}}+{{\rm{H}}}_{2}{\rm{O}}\to {{\rm{CO}}}_{2}+{{\rm{H}}}_{2}$$

22 $${\rm{C}}+{{\rm{CO}}}_{2}\to 2{\rm{CO}}$$

In the studied system the subsequent reaction of the coupling of methane occurs^44^. The formation of radicals (reactions 23 and 24) and ion-radicals is the decisive stage for the consecutive transformations of methane^33^.

23 $${{\rm{CH}}}_{4}+{\rm{e}}\to {{\rm{CH}}}_{3}+{\rm{H}}+{\rm{e}}$$

24 $${{\rm{CH}}}_{4}+{\rm{e}}\to {{\rm{CH}}}_{2}+2{\rm{H}}+{\rm{e}}$$

From CH_3_ and CH_2_ radicals ethane, were formed (25, 26).

25 $${{\rm{CH}}}_{4}+{{\rm{CH}}}_{2}\to {{\rm{C}}}_{2}{{\rm{H}}}_{6}$$

26 $$2{{\rm{CH}}}_{3}\to {{\rm{C}}}_{2}{{\rm{H}}}_{6}$$

Ethylene and ethyne may be generated by the electron-impact ionization and dehydrogenation of C_2_H_6_ (reaction 27–28).

27 $${{\rm{C}}}_{2}{{\rm{H}}}_{6}+{\rm{e}}\to {{\rm{C}}}_{2}{{\rm{H}}}_{5}+{\rm{H}}+{\rm{e}}$$

28 $${{\rm{C}}}_{2}{{\rm{H}}}_{5}+{\rm{e}}\to {{\rm{C}}}_{2}{{\rm{H}}}_{4}+{\rm{H}}+{\rm{e}}$$

The higher C3 and C4 hydrocarbons were generated in subsequent recombination of C_2_H_5_ and CH_3_ radicals (reaction 29–30).

29 $${{\rm{C}}}_{2}{{\rm{H}}}_{5}+{{\rm{CH}}}_{3}\to {{\rm{C}}}_{3}{{\rm{H}}}_{8}$$

30 $$2{{\rm{C}}}_{2}{{\rm{H}}}_{5}\to {{\rm{C}}}_{4}{{\rm{H}}}_{10}$$

A determination of toluene conversion selectivity is very difficult in the CPCS. In this system, a series of reactions run, whose products are benzene and ethyne. This is the intermediate step of toluene oxidation to carbon dioxide and water. These intermediates can also decompose into soot (reactions 15 and 16), which can oxidize to carbon dioxide. In the CPCS methanation of carbons oxides (17, 18) and carbon black hydrogenation (19) can occur. Under these conditions, carbon monoxide can also undergo the water-gas shift reaction (21) or can be formed in the Boudouard’s reaction (22). It should also be added that under these conditions there is also a reaction of carbon dioxide dissociation (5). This causes that the selectivity of the conversion of toluene to individual products is impossible to determine.

## Conclusion

On the basis of the obtained results, it has been found that the gliding discharge is an effective technique for the decomposition of toluene in the pyrolysis gases with low toluene concentrations.

In the CPCS with RANG-19PR (the industrial catalyst for methanation of carbon oxides) a higher toluene conversion was obtained than that in the homogeneous system. A small amount of hydrocarbons C2-C4 was also observed. The presence of Ni** + **NiO/Al_2_O_3_ (RANG19PR) in the plasma zone increased the conversion of toluene and methanation of carbon oxides. The maximum toluene conversion was 89%.

The use of the catalyst increased the calorific value of the outlet gas for a higher power. It was above the minimal level required by engines and turbines. In the CPCS with RANG-19PR, the conversion of toluene was constant within standard error for 40 minutes. The amounts of tars were reduced tenfold.
